# Late discovery of left atrial appendage occluder device embolization: a case report

**DOI:** 10.1186/s12872-020-01589-9

**Published:** 2020-06-22

**Authors:** Mohamad Jihad Mansour, Clément Bénic, Romain Didier, Antoine Noel, Martine Gilard, Jacques Mansourati

**Affiliations:** 1 Division of Cardiology, Lebanese University, Faculty of Medical Sciences, Beirut, Lebanon; 2 Department of Cardiology, University Hospital of Brest, Brest, Cedex 29609 France; 3 Université de Bretagne Occidentale, Brest, France

**Keywords:** Left atrial appendage closure, Watchman device, Atrial fibrillation, Stroke, Echocardiography, Case report

## Abstract

**Background:**

Left atrial appendage (LAA) closure has been well evaluated in the prevention of stroke in patients with atrial fibrillation. Device embolization remains one of the most common complications. To the best of our knowledge, there have been no reports of late discovery of LAA occluder device embolization at 1.5 years after implantation.

**Case presentation:**

We describe the case of a 77-year-old man who underwent uneventful LAA closure. Echocardiography performed the next day showed the device in place. The patient was discharged but was then lost to follow-up. 1.5 years later, he was admitted for ischemic stroke. Transesophageal echocardiography showed the absence of the occluder device in the LAA. Computed tomography scan of the abdomen showed the device in the abdominal aorta. Due to the high cardiovascular risk, the device was kept in place and the patient was treated medically.

**Conclusions:**

Per-procedural and late device embolization are not uncommon. Review of the literature however showed no report of late discovery of device embolization at 1.5 years. Follow-up echocardiography is mandatory for the detection of endothelialization or embolization.

## Background

Several studies have evaluated different left atrial appendage (LAA) occluder devices and demonstrated non-inferiority in stroke prevention compared to warfarin in patients with atrial fibrillation (AF) [1, 2]. Early device embolization remains one of the most common complications, which requires urgent extraction. We herein describe a case of late discovery of an occluder device embolization that was not extracted but rather medically managed.

## Case presentation

A 77-year-old male patient with a medical history significant for permanent AF with a CHA_2_DS_2_-VASC score of 6, ischemic stroke with residual seizure and two hemorrhagic strokes, was referred for LAA closure using a Watchman device (Boston Scientific, Inc., Natick, Massachusetts). LAA morphology and measurements were obtained from cardiac computed tomography (CT) angiography and transesophageal echocardiography (TEE). LAA was bilobed. The maximum width of the ostium was measured at 20 mm. Hence, a 24 mm device was successfully implanted. The device was well aligned with the axis of the LAA. A gentle tug test did not change the device position. The patient remained stable and there were no complications noted during or after the procedure. Transthoracic echocardiography (TTE) performed the next day showed the device in place. The patient was discharged with a scheduled TEE six weeks after the procedure but was lost to follow-up.

**Fig. 1:**
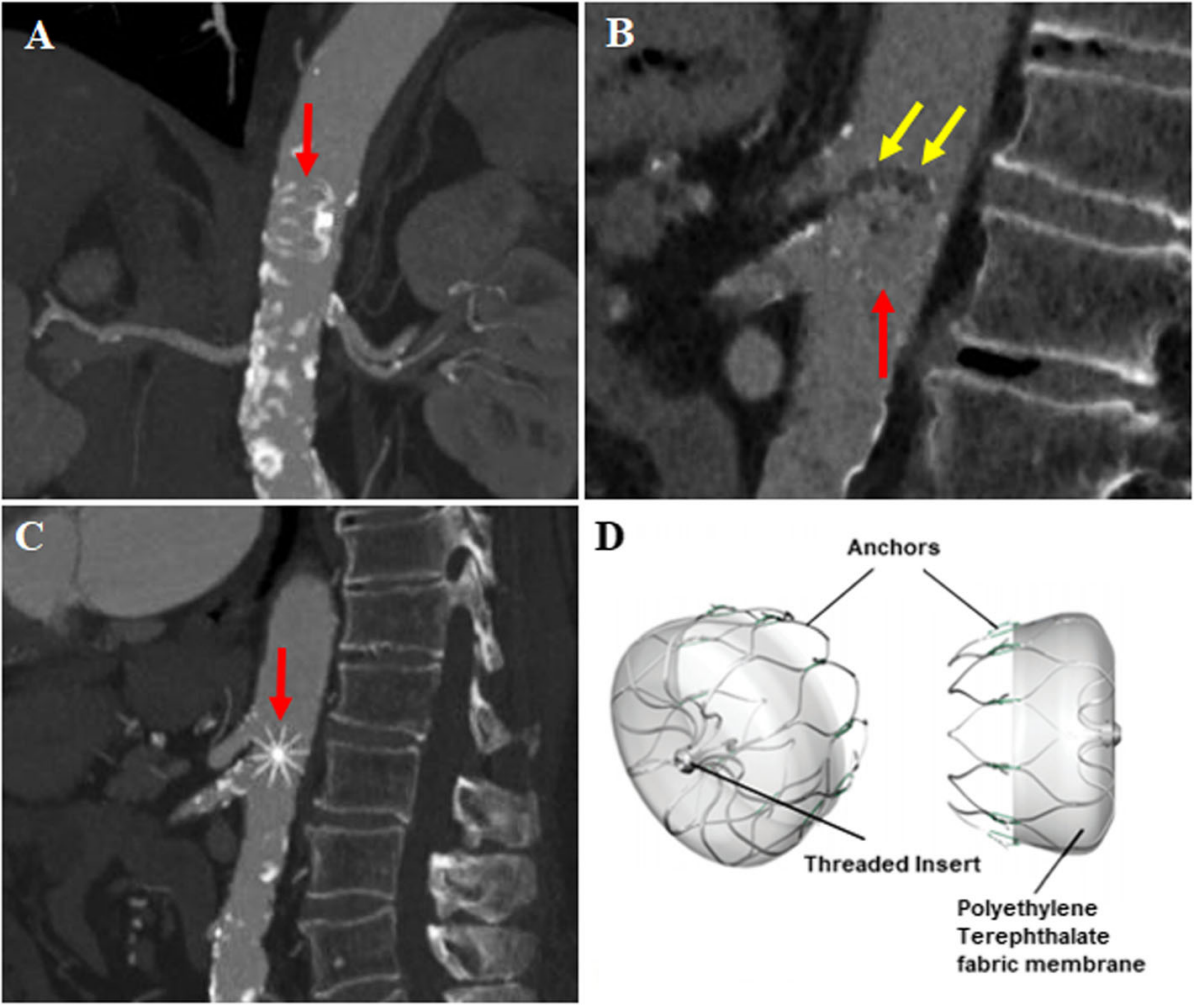
Watchman device (red arrows) located in the abdominal aorta in coronal (**a**), sagittal right (**b**) and sagittal left (**c**) views. Note the mild thrombus formation in the device in panel B (yellow arrows). Panel **d** illustrates the general structure of the Watchman device

1.5 years later, he presented with two new ischemic strokes and unexplained left foot pain. Repeat TTE/TEE showed the absence of the occluder device in the LAA. CT scan of the chest and abdomen showed the device in the abdominal aorta between the ostium of the celiac trunk and the superior mesenteric artery (Fig. 1, Panels A-C). Mild thrombosis was seen in the device at the level of the fabric membrane (Panels B and D). The abdominal aorta was severely calcified (Panels A and C). Due to the high cardiovascular risk, surgical or percutaneous extraction were not done and the device was kept in place. Low dose aspirin was added to his medical treatment. The patient died 3 months later from seizure.

## Discussion and conclusions

Complications of Watchman device implantation are rare, with device embolization rates of 0.6 and 0.7% [1, 2]. Device extraction can be performed either percutaneously via a snare introduced in the femoral artery sheath (e.g., for Watchman device), or surgically (for larger devices) [3]. Percutaneous removal remains the treatment of choice for vascular embolization, particularly in patients with multiple comorbidities and the elderly population. Device embolization risk depends on the operator’s experience, the choice of device size and the final position. Patient related characteristics such as LAA morphology and length, ostium size or unusual morphologies are also important criteria. Per procedural TEE guidance is mandatory, thereby avoiding vigorous tug testing (usually performed for proof of device stability). Nevertheless, aggressive physical movements are not advised before endothelialization [4].

Published articles retrieved from PubMed database included single center/multicenter registries, randomized controlled trials, observational studies, case reports and a systematic review [3–24] (Table 1). Device embolization occurred mostly during the procedure and within 7 days in the described cases. Some cases reported embolization at 45 and 48 days [3, 16, 19]. A study published by Swaans et al. [5] described device embolization 3 months following the procedure. Another case described percutaneous retrieval of an AMPLATZER cardiac plug 6 months after embolization [23]. In a systematic review, Aminian et al. [24] concluded that embolization occurred mostly in the periprocedural period but late embolization was not uncommon. Review of the literature however showed no report of late discovery of device embolization at 1.5 years. Since in the majority of cases device embolization is asymptomatic, patient education for short and long term follow-up is extremely important as there is no way to know the exact timing of device embolization. Hence, in our case, embolization could have occurred earlier but was lately picked up due to loss of follow-up.

**Table 1:** Summary of published data on Watchman device embolization

Reference	Study Design	Number of device embolization	Device size	Device location	Timing	Retrieval Approach
Holmes et al. [2]	Randomized controlled trial (*N* = 269)	2	27 mm	LV	Post procedure day 1	Surgery
Holmes et al. [3]	Randomized non-inferiority trial (*N* = 463)	3	30 mm	LV Thoracic Aorta AA	Intraprocedural 45 days 45 days	Surgery Percutaneous (femoral – snare) Surgery
Sick et al. [4]	Multicenter registry (*N* = 66)	2	NA	NA	Intraprocedural	Percutaneous (femoral – snare)
Swaans et al. [5]	Single center registry (*N* = 30)	1	NA	AA	3 months	Surgery
Reddy et al. [6]	Multicenter registry (*N* = 150)	2	NA	Descending Aorta	Intraprocedural	Percutaneous (femoral – snare)
Matsuo et al. [7]	Single center registry (*N* = 179)	2	NA	AA	Post procedure within 12 h	Percutaneous (femoral – snare)
Pérez Matos et al. [8]	Case report	1	27 mm	LV	Post procedure day 1	Transapical access and pulling catheter
Chopra et al. [9]	Case report	1	34 mm	LA	Post procedure day 1	Transseptal
Vivek et al. [10]	Registry (*N* = 3822)	9	NA	NA	NA	6 surgery 3 Percutaneous
Boersma et al. [11]	Cohort (*N* = 1025)	2	NA	NA	Within 7 days	1 surgery 1 percutaneous
Vivek et al. [12]	RCT (*N* = 707)	3	NA	NA	Early	NA
Pillarisseti et al. [13]	Multicenter observational study (*N* = 478)	1	NA	NA	NA	Surgery
Betts et al. [14]	Multicenter retrospective registry (*N* = 371)	1	NA	NA	Per procedure	NA
Saw et al. [15]	Multicenter experience	1	NA	NA	Early	Percutaneous –Snared
Fanari et al. [16]	Case report	1	21 mm	AA	48 days	Percutaneous
Gabriels et al. [17]	Case report	1	24 mm	LA	Intraprocedural	Percutaneous – transseptal
Fastner et al. [18]	Case report	1	NA	LA	Intraprocedural	Percutaneous
Hai Deng et al. [19]	Case report	1	30 mm	Aortic arch	45 days	Percutaneous – snared
Stollberger et al. [20]	Case report	1	30 mm	LV	Periprocedural	Surgery
Barth et al. [21]	Case Report	2	24 mm 21 mm	LA Descending Aorta	Periprocedural	Percutaneous – transseptal Percutaneous –Snared
Bôsche et al. [22]	Single center prospective study	1	NA	NA	Within 7 days	Percutaneous
Obeid et al. [23]	Case report	1	24 mm	LA	6 months	Percutaneous
Aminian et al. [24]	Systematic Review	21	NA	9 Aorta 9 LV 3 LA	Until 90 days	Surgical Percutaneous

*AA* abdominal aorta, *LA* left atrium, *LV* left ventricle, *NA* not applicable

We report a unique case of late discovery of LAA occluder device embolization in the abdominal aorta. Per procedural and follow-up echocardiography is crucial for the detection of device endothelialization or embolization.

## Data Availability

Not applicable.

## Abbreviations

AF: Atrial fibrillation
CT: Computed tomography
LAA: Left atrial appendage
TEE: Transesophageal echocardiography
TTE: Transthoracic echocardiography
